# Effects of tildrakizumab on circulating T cells in autoreactive psoriatic patients

**DOI:** 10.3389/fimmu.2026.1795117

**Published:** 2026-04-10

**Authors:** Paola Facheris, Rebecca Favaro, Mario Valenti, Antonio Costanzo, Riccardo G. Borroni

**Affiliations:** 1 Dermatology Unit, Humanitas Research Hospital - IRCCS, Rozzano, Milan, Italy; 2 Department of Biomedical Sciences, Humanitas University, Pieve Emanuele, Milan, Italy

**Keywords:** ADAMTSL5, autoreactivity, inflammation, LL37, psoriasis, T cells, tildrakizumab

## Abstract

**Introduction:**

T cell autoreactivity against the autoantigens LL37 and ADAMTSL5 contributes to inflammation in a subset of psoriatic patients. The immunological mechanisms underlying the treatment response in patients showing autoreactivity remain incompletely understood in clinical settings. This study investigated the impact of tildrakizumab on circulating immune populations and on autoantigen-specific T cell reactivity.

**Methods:**

Patients with moderate-to-severe psoriasis were enrolled and treated with tildrakizumab according to clinical practice. T cells autoreactivity to LL37 and ADAMTSL5 was assessed at baseline using a stimulation index (SI>2). Longitudinal changes in circulating T cell subsets were analyzed using multiparametric flow cytometry.

**Results:**

Twenty of 38 psoriatic patients (52.6%) displayed circulating autoreactive T cells (SI>2) to LL37 and/or ADAMTSL5, compared with 5 of 33 healthy donors (15.2%). Baseline frequencies of Ki67^+^CD4^+^/CD8^+^, and IL-17^+^CD4^+^/CD8^+^ T cell populations positively correlated with PASI. Stimulation of PBMCs with LL37 or ADAMTSL5 induced Th17- and Th1-related cytokines, but not Th2-related cytokines. During treatment, tildrakizumab significantly modulated multiple circulating T cell subsets, including Ki67^+^CD4^+^, Ki67^+^CD8^+^, Ki67^+^Th17, Th17, and Ki67^+^Tregs, and markedly reduced autoreactivity to both autoantigens. Notably, clinical improvement strongly correlated with reductions in Ki67^+^CD4^+^ and Ki67^+^CD8^+^ T cell frequencies. LL37-reactive patients exhibited a poorer clinical response at weeks 40 and 52.

**Conclusions:**

In autoreactive patients, tildrakizumab effectively modulated circulating T cell proliferation and reduced autoantigen-specific responses over time. LL37-reactive patients showed distinct long-term responses, suggesting that autoantigen-reactive subjects may represent a more treatment-challenging subgroup within the psoriatic population.

## 1 Introduction

Psoriasis is a chronic inflammatory disease affecting the skin. In psoriatic lesions, cytokines such as IL-23 and IL-17 play a key role in sustaining chronic skin inflammation (1). Structurally, IL-23 is a heterodimer composed of the p19 subunit (IL-23p19) and the p40 subunit, the latter also being part of IL-12 (2, 3). IL-23 is essential for the expansion of memory T cells, the regulation of antibody production, and induction of IFN-γ, and it is a key driver of Th17-cell maturation and proliferation. Th17 cells secrete IL-17 and IL-22, which contribute to host defense against infections but are also involved in chronic inflammatory diseases such as psoriasis (1, 4, 5). The identification of the role of IL-17 and IL-23 enabled the development of biologic drugs for chronic plaque psoriasis (6). Following phase III studies showing 75% skin clearance (75% reduction in Psoriasis Area and Severity Index, PASI 75) in 61%-66% of patients by week 12, and in 73%-79% at week 28 after only three doses (6, 7), tildrakizumab, a humanized IgG1/κ monoclonal antibody that specifically targets the IL-23p19 (8), was licensed for the treatment of adults with moderate-to-severe chronic plaque psoriasis.

In approximately two-thirds of patients with moderate-to-severe psoriasis, inflammation is also sustained by immune responses to skin autoantigens, such as cathelicidin (LL37) (9) and ADAMTSL5 (disintegrin and metalloproteinase with thrombospondin motifs 5) (10). LL37-autoreactive T cells can display either a CD8^+^ or CD4^+^ phenotype and are rare in healthy individuals (9). Whether the inflammatory profile of autoreactive subjects differs from that of non-autoreactive patients, and how this influences clinical responses, remains unclear. A recent study found that circulating T cell reactivity to both LL37 and ADAMTSL5 was associated with a suboptimal clinical response to risankizumab, another monoclonal antibody targeting the IL-23 p19 subunit (11). In contrast, reactivity to either LL37 or ADAMTSL5 alone did not affect clinical outcomes (11). Furthermore, circulating T cell populations were effectively modulated by risankizumab in non-reactive and single-reactive subjects, but not in those exhibiting dual autoreactivity to both LL37 and ADAMTSL5 (11). In this study, we aim to investigate how IL-23 blockade by tildrakizumab influences T cell responses in autoreactive and non-autoreactive psoriatic patients.

## 2 Methods

### 2.1 Study population

Consecutive patients affected by moderate-to-severe psoriasis for at least 6 months, not receiving systemic treatment and candidate for biologic therapy referred to the Dermatology Unit of Humanitas Research Hospital IRCCS in Rozzano (MI), Italy, were enrolled between January 2022 and July 2024. All patients received tildrakizumab treatment as per summary of product characteristics. Healthy donors (HD) with no personal history of skin diseases served as controls. Blood samples were collected at baseline and at weeks 16, 28, and 52. PASI scores were recorded at baseline and at weeks 16, 28, 40, and 52. This study was conducted in accordance with the Note for Guidance on Good Clinical Practice (Humanitas ICH Harmonized Tripartite Guideline E6(R1)); the general guideline indicated in the Declaration of Helsinki, and all applicable regulatory requirements. The study was approved by the Institutional Review Board (no. 3083). Written informed consent was obtained from all participants before enrolment.

### 2.2 Peripheral blood mononuclear cells (PBMCs) isolation and analysis.

Peripheral blood was collected after venipuncture in heparin-containing tubes. Peripheral blood mononuclear cells (PBMCs) were isolated and stimulated as previously described (11). Reactivity within CD4^+^ and CD8^+^ cells to LL37 or ADAMTSL5 was expressed by stimulation index (SI) of proliferation. This was calculated as the ratio between Ki67^+^ T-cells in antigen-stimulated PBMCs and Ki67^+^ T-cells in non-stimulated PBMCs of the same subject after 6 days of *in vitro* culture. The frequency of the evaluated Ki67^+^ population is detailed in the gating strategy (**Supplementary Figure 1**). Statistical analyses were performed according to the defined population hierarchy, and these values were used to calculate the stimulation index (SI). Each proliferation assay was conducted as previously described (11), with two replicate wells per condition; the mean value was used for data representation and statistical analysis. Immunostaining was performed with Zombie Aqua™ Fixable Viability Kit (Biolegend 77143), and human surface markers α-CD3(UCHT1)-PERCPCY5.5 (Biolegend-300430), α-CD4(RPA-T4)–FITC (Biolegend-300538), α-CD8(RPA-T8)-BV421 (BD-562428) and/or α-CD25(2A3)-BV421 (BD-564033); internal markers: α-Ki67(B56) AF700 (BD-561277), α-IL-17A (BL168)-PE (Biolegend-B255234), and α-FOXP3(259D/C7)-AF647 (BD-560045) after FOXP3/Transcription Factor Fixation/Permeabilization (e-bioscience-00-5521-0). To test ILs, cells were treated for 2 hours (37 °C and 5% CO_2_) with Cell activation cocktail without Brefeldin (423302 Biolegend) and Golgi plug (555029 BD) in complete medium. All stained-cell samples were acquired on a BD LSRFortessa and analyzed with DIVA software (BD). The gating strategy to determine SI and phenotyping of Th17, Tc17 and Tregs were described in a previous works (11) and are reported in **Supplementary Figure 1**. Supernatants of PBMCs stimulated with antigens and controls were collected and frozen at -20 °C until further analysis. Cytokine analysis was performed with ProcartaPlex™ Human High Sensitivity Panel, 9plex (EPXS090-12199-901) run in Bio-RAD Bio-Plex 200 Systems.

### 2.3 Statistical analysis

Continuous variables are presented as mean ± standard error of the mean (SEM) or as Min to Max box-and-whisker plot with points, as indicated. Data distribution was assessed using the D’Agostino–Pearson omnibus normality test prior to statistical analysis. Comparisons between two independent groups were performed using unpaired T-test or the Mann-Whitney *U* test. Longitudinal comparisons within the same subjects were analyzed using the Friedman test followed by Dunn’s *post hoc* test. Correlations between variables were assessed using Pearson’s or Spearman correlation coefficient (two-tailed). Statistical analyses were performed using GraphPad Prism version 7.1 (GraphPad Software, San Diego, CA, USA). A p-value <0.05 was considered statistically significant. Missing data at specific patient time points (resulting from missed visits or failure to obtain blood samples) were handled using the Last Observation Carried Forward (LOCF) method.

## 3 Results

### 3.1 Psoriatic patients show higher reactivity to autoantigens

Thirty-eight patients were included. At baseline, stimulation with either LL37 or ADAMTSL5 induced a higher proliferative response (Stimulation Index, SI), in CD8^+^ T cells from psoriatic patients compared with HD (LL37 p=0.0006, ADAMTSL5 p=0.0396; **Supplementary Figure 2**). In CD4^+^ cells, only LL37 elicited a significantly higher SI compared to HD (p=0.0198, **Supplementary Figure 2**).

Using an SI > 2 threshold to define autoreactivity, as previously established by Lande et al. (9, 12), we found that 20 of 38 patients (52.6%) exhibited circulating T-cell autoreactivity (SI > 2) against LL37 and/or ADAMTSL5, compared with 5 of 33 healthy donors (15.2%), confirming a significantly higher prevalence of autoreactivity in psoriasis (p = 0.0012). Patients were defined as autoreactive when an SI > 2 was observed in CD4^+^ or CD8^+^ T cells following stimulation with at least one of the tested antigens. Among autoreactive patients, 7 (35%) were LL37-reactive, 7 (35%) were ADAMTSL5-reactive, and 6 (30%) showed reactivity to both autoantigens. Demographic characteristics of these subjects are summarized in **Supplementary Table 1**.

We then assessed whether localization of psoriatic lesions differed according to autoreactivity status. Autoreactive patients showed significantly more frequent involvement of sensitive or “difficult-to-treat” areas (scalp, face, genitalia and flexural sites) compared with non-reactive subjects (90%, 18/20 vs. 44%, 8/18; p=0.004).

### 3.2 Psoriatic patients show higher baseline Th17, Tc17 and proliferating T cells, correlating with disease severity

**Figure 1:**
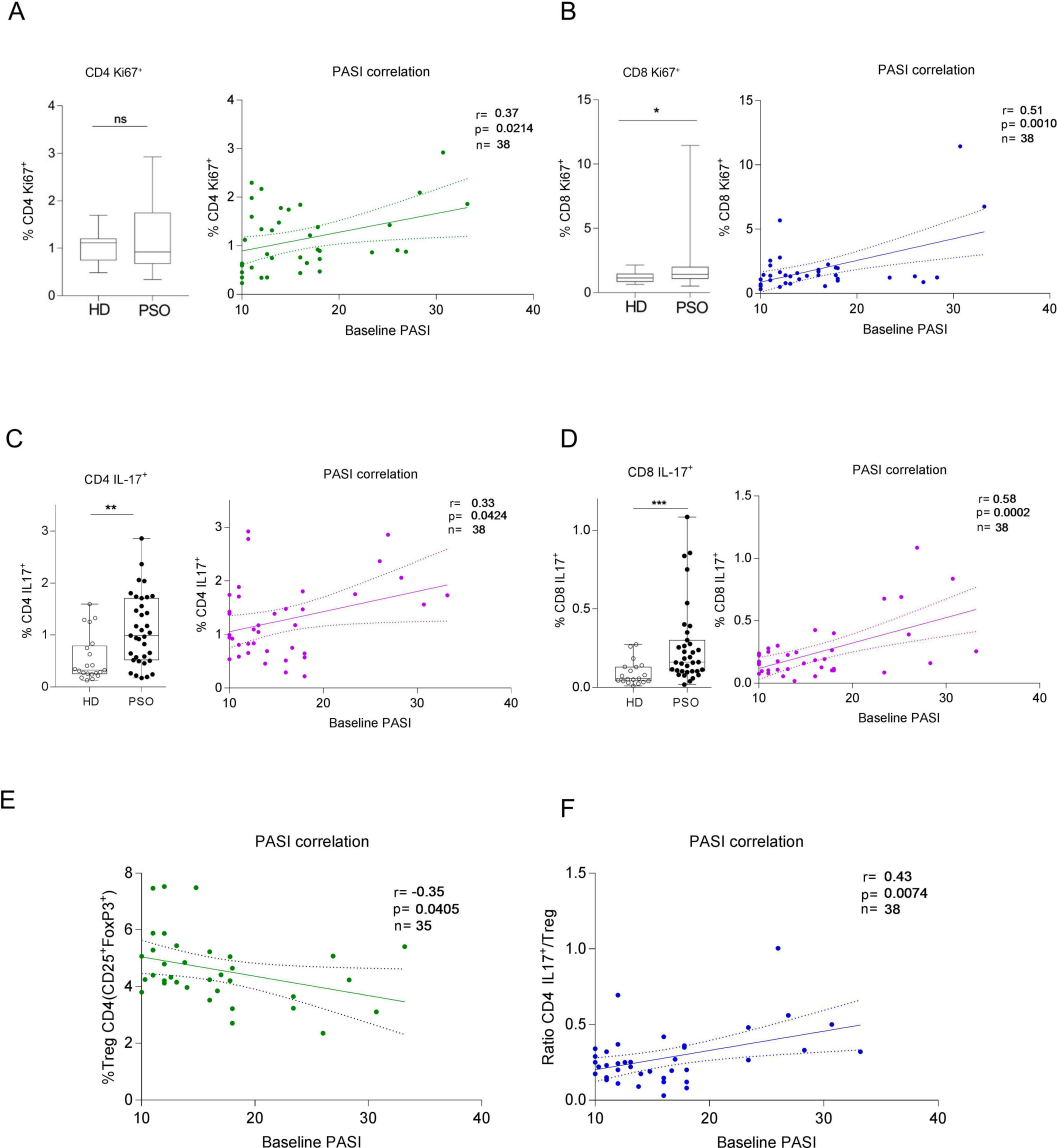
Baseline frequencies of proliferating T cells, Th17 cells, and Tc17 cells correlate with disease severity in psoriatic patients. **(A)** Frequency of Ki67^+^CD4^+^ T cells at baseline did not significantly differ compared with HD, but positively correlated with PASI (r=0.37, p=0.0214). **(B)** Frequency of Ki67^+^CD8^+^ T cells at baseline was significantly higher compared with HD and positively correlated with PASI (r=0.51, p=0.001). **(C)** Th17 cell frequency was significantly higher in psoriatic patients compared with HD (Unpaired t-test p=0.0016) and positively correlated with PASI (r=0.33, p=0.04). **(D)** Tc17 cell frequency was significantly higher in psoriatic patients compared with HD (Mann-Whitney test p=0.0003) and positively correlated with PASI (r=0.58, p=0.0001). **(E)** Baseline Treg frequencies inversely correlated with PASI (r=–0.35, p=0.04). **(F)** The baseline Th17/Treg ratio positively correlated with PASI (r=0.43, p=0.005). Correlations were assessed using Pearson’s correlation coefficient. * p<0.05; ** p<0.01; *** p<0.001; ns, not significant.

We first wanted to characterized proliferating and psoriasis-relevant effector subsets (Ki67^+^ T cells, Th17, Tc17, Treg) at baseline and evaluate their association with disease severity. At baseline, we observed a significantly higher frequency of Ki67+CD8+ T cells in patients compared to healthy donors (HD), whereas the frequency of Ki67+CD4+T cells did not differ significantly between groups. The frequency of both Ki67^+^CD4^+^and Ki67^+^CD8^+^ correlated with baseline PASI (r=0.37, p=0.0214 and r=0.51, p=0.001, respectively; **Figures 1A, B**).

Psoriatic patients exhibited higher frequencies of circulating Th17 and Tc17 cells compared with HD (1.10±0.68 vs 0.55±0.45, p=0.0012; and 0.26±0.25 vs 0.09±0.077, p=0.0003, respectively, **Figures 1C, D**). Their frequencies also correlated with disease severity (r=0.33, p=0.04; and r=0.58, p=0.0001, respectively, **Figures 1C, D**). In addition, baseline PASI negatively correlated with the frequency of the Treg population at baseline (r=-0.35, p=0.04; **Figure 1E**), while showing a positive correlation with the Th17-to-Treg ratio (Th17/Treg; r=0.43, p=0.005; **Figure 1F**).

### 3.3 LL37 and ADAMTSL5 induce the release of Th17- and Th1-related cytokines

To investigate the role of LL37 and ADAMTSL5 in the release of psoriasis-related cytokines, supernatants from LL37- or ADAMTSL5-stimulated PBMCs of reactive (SI>2) subjects were analyzed for the presence of inflammatory cytokines (IFN-γ IL-1β, IL-2, IL-4, IL-6, IL-10, IL-12p70, IL-17A, TNF-α). Significantly higher levels of psoriasis-associated cytokines, namely IL-17A, IFN-γ, and TNF-α, were detected compared with control supernatants (“scramble” peptide or no stimulation, **Supplementary Figure 3**). IL-6 release was also induced by exposure to both antigens, whereas IL-1β secretion was observed only following LL37 stimulation (**Supplementary Figure 3**). In contrast, IL-2, IL-4, IL-10, and IL-12p70 were undetectable (data not shown). These findings confirm that LL37 and ADAMTSL5 are sufficient to induce Th17- and Th1-related cytokine production in autoreactive subjects. Notably, none of these cytokines were detectable in non-reactive (SI<2) subjects, further supporting the specificity of LL37- and ADAMTSL5-driven activation (data not shown).

### 3.4 Tildrakizumab modulates the frequency of circulating T cell subsets and their response to psoriasis autoantigens

After the characterization of circulating immune cell populations at baseline, and confirmation that exposure to LL37 and ADAMTSL5 induces the release of psoriasis-related cytokines in autoreactive subjects, we next investigated the modulation of circulating T cell subsets during tildrakizumab treatment.

**Figure 2:**
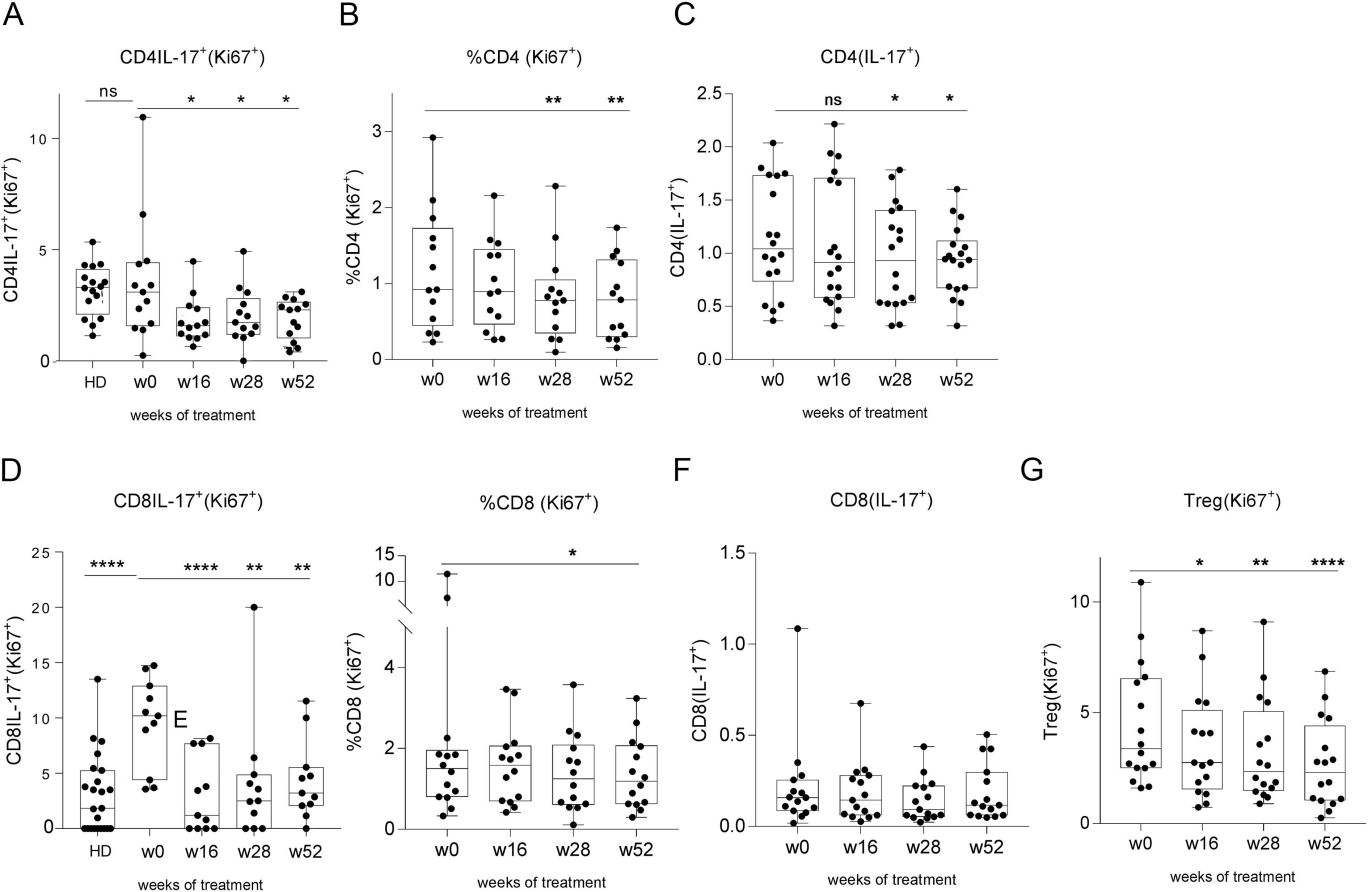
Tildrakizumab reduces the frequency of circulating inflammatory T cell populations. Tildrakizumab modulates specific T cell populations: **(A)** Ki67^+^ Th17 (Ki67^+^CD4^+^IL-17^+^) cell frequency was similar in psoriatic patients compared with HD (Mann Whitney), but significantly decreased from week 16; **(B)** Ki67^+^CD4^+^ cells significantly decreased from week 28; **(C)** Th17 (CD4^+^IL17^+^) significantly decreased from week 28; **(D)** Ki67^+^Tc17 (Ki67^+^CD8^+^IL17^+^) cell frequencies were higher in psoriatic patients compared with HD (Mann Whitney test <0.0001), and significantly decreased from week 16; **(E)** Ki67^+^CD8^+^ cells significantly decreased at week 28; **(F)** Tc17 decreased at week 28, but did not reach statistical significance. **(G)** Ki67^+^Treg cells significantly decreased from week 16; Data are shown as min-to-max box-and-whisker plots with individual points. Multiple comparisons were performed using the Friedman test followed by Dunn’s uncorrected *post hoc* test. * p<0.05; ** p<0.01; *** p<0.001; ns, not significant.

A significant reduction in Ki67^+^Th17 cells was already evident at week 16 (**Figure 2A**), followed by a significant decline in both Ki67^+^CD4^+^ T cells and total Th17 cells at week 28 (**Figures 2B, C**). Ki67^+^Tc17 cells also showed a significant decrease beginning at week 16 (**Figure 2D**), whereas Ki67^+^CD8^+^ T cells exhibited a reduction only at week 28 (**Figure 2E**). A decline in the frequency of circulating Tc17 cells was observed at week 28, although not statistically significant (**Figure 2F**).

Ki67^+^Tregs also decreased over the course of treatment (**Figure 2G**), whereas the overall frequency of Tregs remained stable (data not shown), suggesting that tildrakizumab primarily modulates proliferative activity rather than the absolute size of the regulatory T cell compartment.

**Figure 3:**
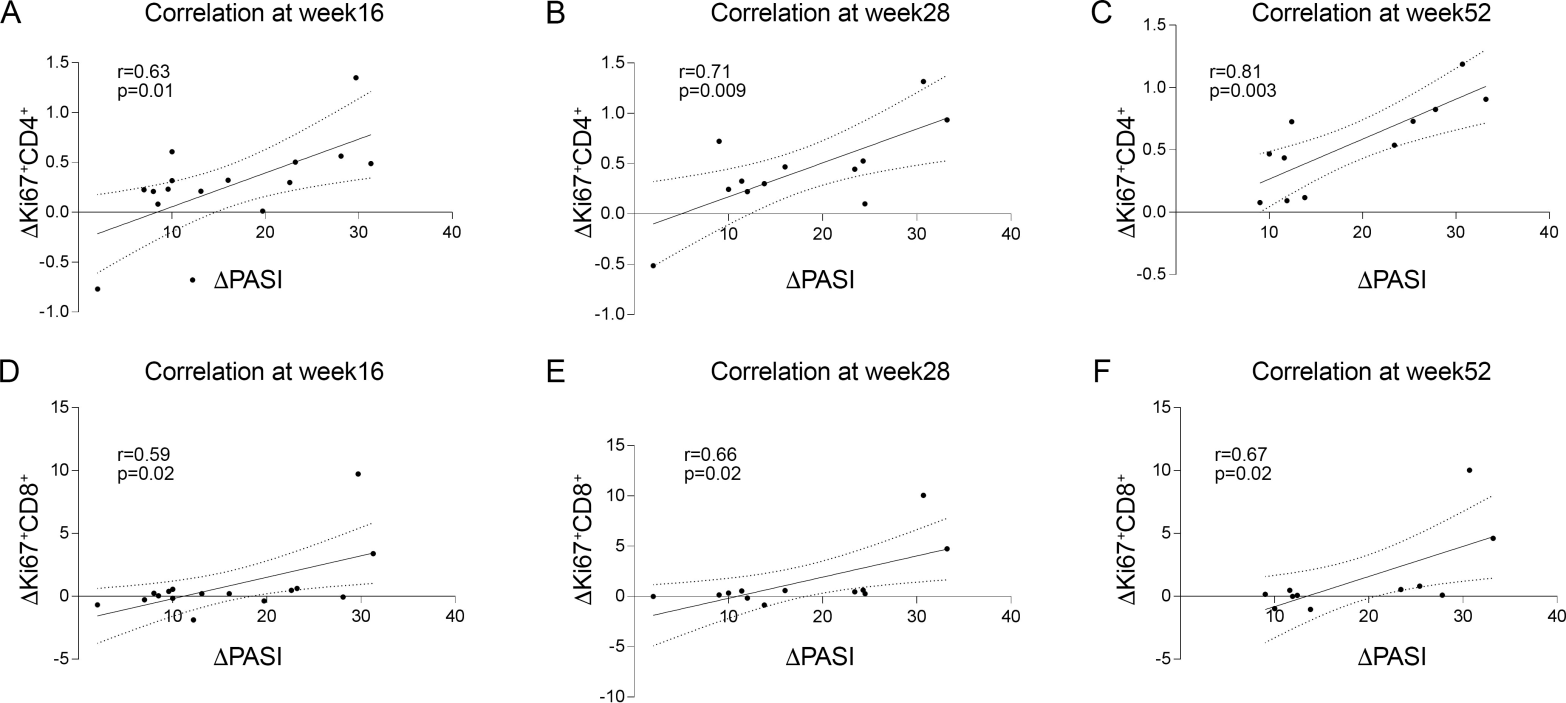
Correlation between PASI reduction and decreases in Ki67^+^CD4^+^ and Ki67^+^CD8^+^ T cells during tildrakizumab treatment. Pearson correlation analyses between changes in PASI score (ΔPASI) and changes in the frequency of proliferating T cells during treatment. Decrease in PASI positively correlated with the decreased in Ki67^+^CD4^+^at week 16 [**(A)** r=0.63, p=0.01], week 28 [**(B)** r=0.71, p=0.009] and week 52 [**(C)** r=0.81, p=0.003]. Decrease in PASI positively correlated with the decreased in Ki67^+^CD8^+^at week16 [**(D)** r=0.59, p=0.02], week 28 [**(E)** r=0.66, p=0.02] and week 52 [**(F)** r=0.67, p=0.02]. Statistical significance was defined as p<0.05 (two-tailed).

The reduction in proliferating T cells correlated with clinical improvement, as the decrease in Ki67^+^CD4^+^ and Ki67^+^CD8^+^ T cell frequency positively correlated with PASI reduction at different timepoints (**Figure 3**).

**Figure 4:**
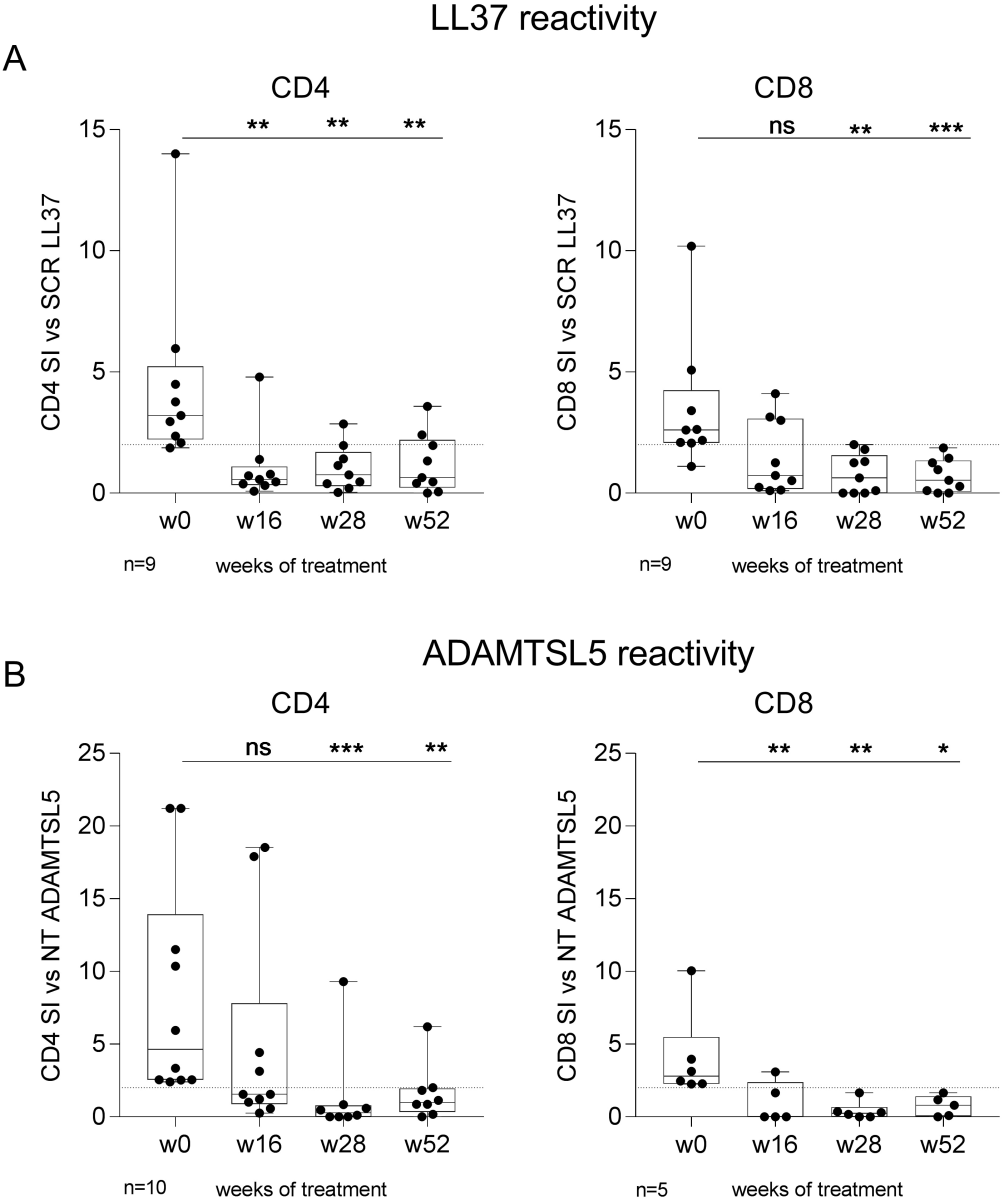
Tildrakizumab reduces autoreactive CD4^+^ and CD8^+^ T cell responses to psoriasis autoantigens. Autoreactive T cell proliferation was assessed by stimulation index (SI) following stimulation with LL37 or ADAMTSL5 at different time points during treatment. **(A)** LL37-induced proliferation of CD4^+^ T cells decreased from week 16. **(B)** LL37-induced proliferation of CD8^+^ T cells decreased from week 28. **(C)** ADAMTSL5-induced proliferation of CD4^+^ T cells decreased from week 28. **(D)** ADAMTSL5-induced proliferation of CD8^+^ T cells decreased from week 16. Data are shown as min-to-max box-and-whisker plots with individual points. Multiple comparisons were performed using the Friedman test followed by Dunn’s uncorrected *post hoc* test. * p<0.05; ** p<0.01; *** p<0.001; ns, not significant.

To evaluate the effect of tildrakizumab on circulating autoreactive CD4^+^ and CD8^+^ T cells, we assessed the SI following LL37- or ADAMTSL5-induced activation at multiple timepoints during treatment (**Figure 4**). Tildrakizumab reduced LL37-induced proliferation of CD4^+^ T cells from week 16 onward and in CD8^+^ T cells by week 28 (**Figures 4A, B**). The drug also attenuated ADAMTSL5-induced proliferation, with a reduction observed in CD4^+^ T cells at week 28 and in CD8^+^ T cells as early as week 16 (**Figures 4B, C**).

### 3.5 Reactive subjects demonstrate a distinct response to treatment

Improvement of psoriasis during tildrakizumab therapy in our cohort was consistent with outcomes shown in randomized, placebo-controlled trials and “real-world” studies (8, 14) (**Supplementary Table 2**). However, when stratifying patients according to autoreactivity status at baseline, reactive subjects exhibited inferior PASI responses at week 40 compared with non-reactive subjects (**Supplementary Table 2**). Notably, LL37-reactive individuals demonstrated a significantly lower clinical response to tildrakizumab than both double-reactive and non-reactive subjects at weeks 40 and 52 (**Supplementary Figure 4A**). Although the proportion of patients achieving PASI90 response was numerically inferior among reactive subjects at both week 40 and week 52, only PASI75 response was significantly reduced at week 40 compared to non-reactive subjects (**Supplementary Figures 4B, C**).

## 4 Discussion

Reactivity of T cells to autoantigens is recognized as an important component of psoriatic inflammation (9). In our cohort, we first evaluated the effect of autoreactivity on the baseline inflammatory T cell population and on the cytokine profile. Exposure of CD4^+^ and CD8^+^ T cells to LL37 or ADAMTSL5 triggered significantly higher proliferative responses in psoriatic patients compared with healthy donors. Autoreactivity (SI>2) toward either autoantigen was detected in 53% of patients, consistent with previously reported frequencies (9). Interestingly, reactive subjects demonstrated a higher involvement of difficult-to-treat areas compared to the general psoriatic population (45–56%) (13). Furthermore, stimulation with LL37 or ADAMTSL5 induced robust Th1/Th17 cytokine production (IL-17A, IFN-γ, TNF-α), confirming that autoantigen-induced T cell activation preferentially drives the IL-17-mediated inflammatory pathway, but not other pathways such as Th2 inflammation. At baseline, the frequencies of proliferating Ki67^+^CD4^+^ and Ki67^+^CD8^+^ T cells, as well as Th17 and Tc17 subsets, correlated positively with psoriasis severity, while Treg frequencies correlated negatively. The Th17/Treg ratio also correlated with PASI, indicating a profound imbalance toward IL-17-driven inflammation in more severe disease (11). Taken together, our findings strengthen the evidence implicating LL37 and ADAMTSL5 in the immunopathogenesis of psoriasis and indicate that T cell autoreactivity may represent an additional pathogenic driver.

Given the key role of IL-23 in Th17 and Tc17 differentiation, we expected that therapeutic blockade of IL-23p19 by tildrakizumab would modulate IL-17-secreting T cell populations. Indeed, we observed an early decline in proliferating Ki67^+^Th17 cells, followed by a broader decrease in circulating Th17 cells and Ki67^+^CD4^+^ T cells by week 28. Proliferating Tc17 cells (Ki67^+^Tc17) also diminished by week 16, although total Tc17 frequencies were less affected over time. Ki67^+^CD8^+^ T cells decreased at week 28, and a reduction in Ki67^+^ Tregs was likewise observed, indicating a more global attenuation of proliferative immune activity under IL-23p19 inhibition. In parallel, tildrakizumab markedly decreased the frequency of autoreactive T cells responding to LL37 and ADAMTSL5 autoantigens as early as week 16, demonstrating a suppressive effect on autoantigen-driven T cell activation.

Interestingly, the decline in IL-17-producing and proliferating T cell populations tightly correlated with clinical improvement. In particular, decrease of Ki67^+^CD4^+^ and Ki67^+^CD8^+^ T cell frequencies showed a strong positive correlation with PASI reduction across multiple time points. These results indicate that the clinical efficacy of tildrakizumab is related to its ability to suppress IL-23-dependent activation and proliferation of T cells. To date, most translational studies on biologic therapies in psoriasis, including anti-TNF-α, anti-IL-17 or anti-IL-23 monoclonal antibodies, have focused on histologic changes in lesional skin, typically showing reduction in keratinocyte proliferation (Ki67 expression) and overall inflammatory infiltrates rather than on Ki67 expression in circulating T cell subsets (14–16). Similarly, studies on the IL-23p19 inhibitors guselkumab and risankizumab have mostly focused on histopathological improvement of lesional skin and IL-23/IL-17 axis signatures, with very limited evaluation of proliferating circulating T cells (17–19). Except for our group’s recent work showing that risankizumab reduces Ki67^+^CD4^+^ and Ki67^+^CD8^+^ T cell frequencies, we were unable to identify studies addressing treatment-induced changes in Ki67^+^ T cell subsets in peripheral blood or linking these changes to clinical response (20). In fact, the changes we observed are consistent with our previous findings with risankizumab, which markedly reduced Th17 and Tc17 populations and decreased autoreactivity to LL37, although ADAMTSL5-specific responses were less modulated (11). In that study, double-reactive patients showed limited immunological modulation, a finding we were unable to confirm here due to the small number of double-reactive subjects treated with tildrakizumab. Although the analysis of circulating rather than skin-resident cells may not capture lesional skin dynamics completely, the strong correlation between systemic T cell activation and disease severity suggests that circulating autoreactive and IL-17-producing T cells reflect the inflammatory burden in psoriasis.

Clinically, reactive patients showed diminished PASI responses at week 40, and specifically LL37-reactive individuals demonstrated reduced responses at weeks 40 and 52, suggesting that distinct autoreactive signatures may influence treatment efficacy. Longer prospective studies might shed light on the dynamics of autoreactivity modulation by tildrakizumab beyond the first year of treatment.

Overall, our findings demonstrate that IL-23 blockade effectively reduces IL-17-producing, proliferating, and autoreactive T cell subsets, and that this immunological modulation correlates with clinical improvement. These results strengthen the rationale for targeting the IL-23/IL-17 axis and highlight the potential relevance of T cell autoreactivity profiling in influencing treatment responses.

## Data Availability

The datasets presented in this article are not readily available because they contain sensitive personal data and access is restricted to protect patient privacy. Requests to access the datasets should be directed to Rebecca Favaro, rebecca.favaro@humanitasresearch.it.
