# Supplementary material for: Effects of tildrakizumab on circulating T cells in autoreactive psoriatic patients
Source: Front Immunol. 2026 Apr 10;17:1795117. doi: 10.3389/fimmu.2026.1795117 (PMC13106394; doi:10.3389/fimmu.2026.1795117)
Supplement: Supplementary file 1 [file DataSheet1.pdf]

Supplementary Material

Effects of tildrakizumab on circulating T cells in autoreactive psoriatic patients

Paola Facheris[1]†, Rebecca Favaro[2]†, Mario Valenti[1,2], Antonio Costanzo[1,2], Riccardo G. Borroni[1,2]*,

[1] Dermatology Unit, Humanitas Research Hospital - IRCCS, Rozzano, Milan, Italy

[2] Department of Biomedical Sciences, Humanitas University, Via Rita Levi Montalcini 4, 20072 Pieve Emanuele, Milan, Italy

[†]These authors share first authorship.

Supplementary Figure 1, 2, 3,4.

Supplementary Table 1, 2.

Supplementary Figure 1.

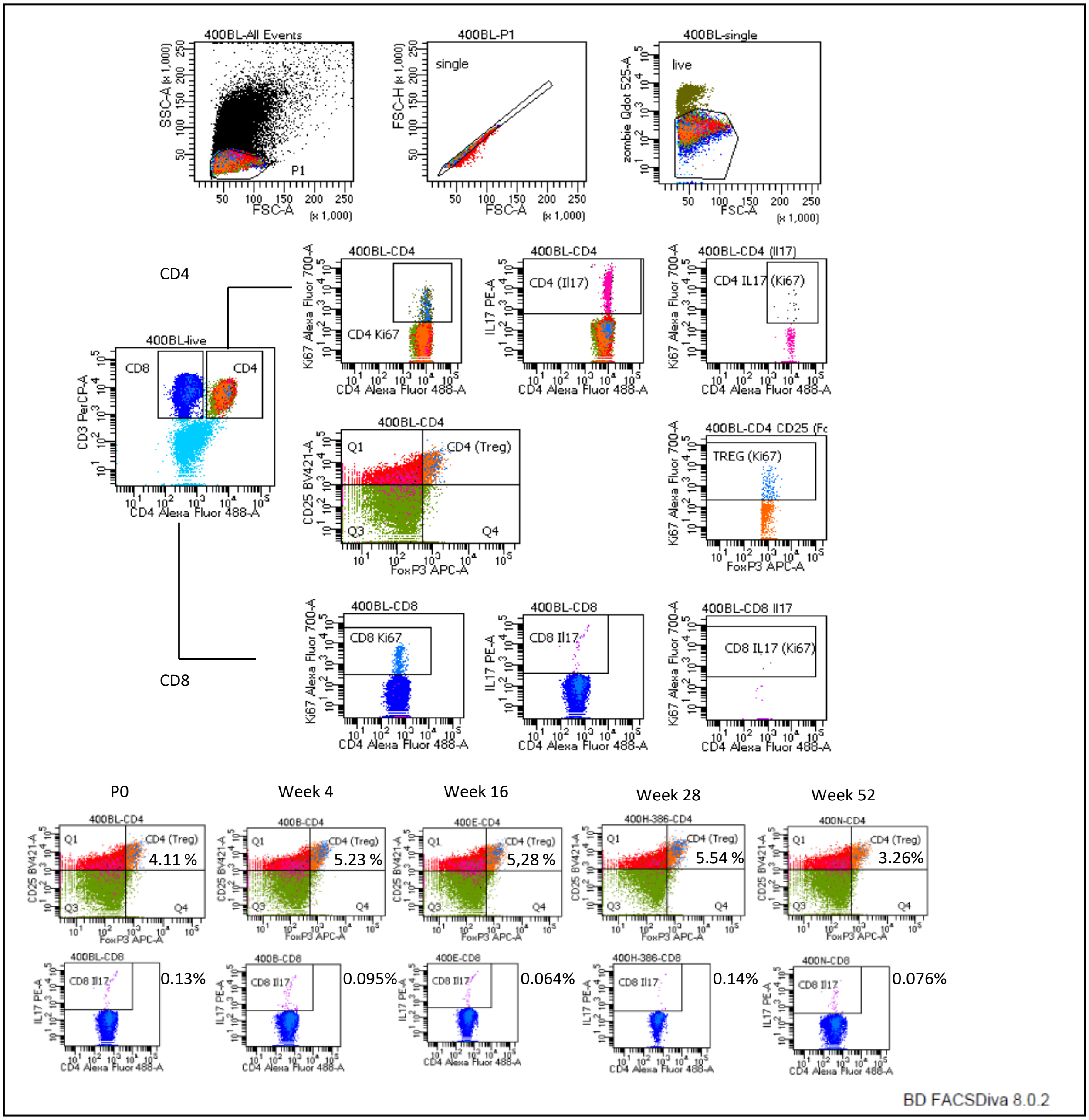

Supplementary Figure 1: Gating strategy to phenotype T cells.

Gating strategy to calculate the frequency of different cellular phenotypes: $CD4^+Ki67^+$, CD^+4IL17^+ CD^+4IL17^+($Ki67^+$), $CD4^+$Treg($CD4^+CD25^+FoxP3^+$), Treg$Ki67^+$ and $CD8^+$ $Ki67^+$, $CD8^+$ $IL17^+$ $CD8^+IL17^+$($Ki67^+$) and examples of T-cell Treg and $CD8^+IL17^+$ at different timepoints. FACS and analysis are performed with BD FACS DIVA 8.02 software.

Supplementary Figure 2.

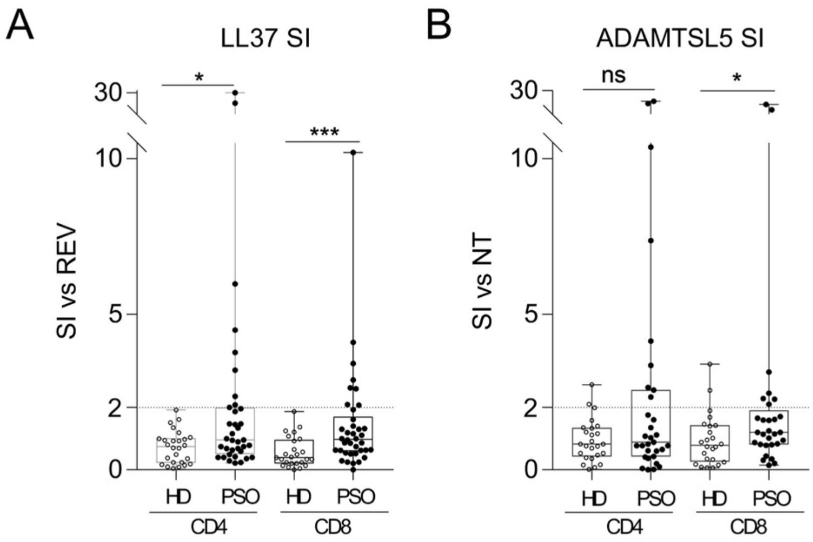

Supplementary Figure 2: Stimulation Index in psoriatic subjects versus healthy donors (HD).

A. Stimulation with LL37 induced a higher proliferating response compared to HD, calculated as SI (Stimulation Index), in $CD4^+$ (p=0.0198) and $CD8^+$ (p=0.0006) T cells.
B. ADAMTSL5 induced a higher proliferating response compared to healthy donors in $CD8^+$ T cells (p=0.0396), but not in $CD4^+$.
Statistical analysis was performed using a two-tailed Mann-Whitney test. A p-value <0.05 was considered statistically significant.

* p<0.05; ** p<0.01; *** p<0.001; ns, not significant.

Supplementary Figure 3.

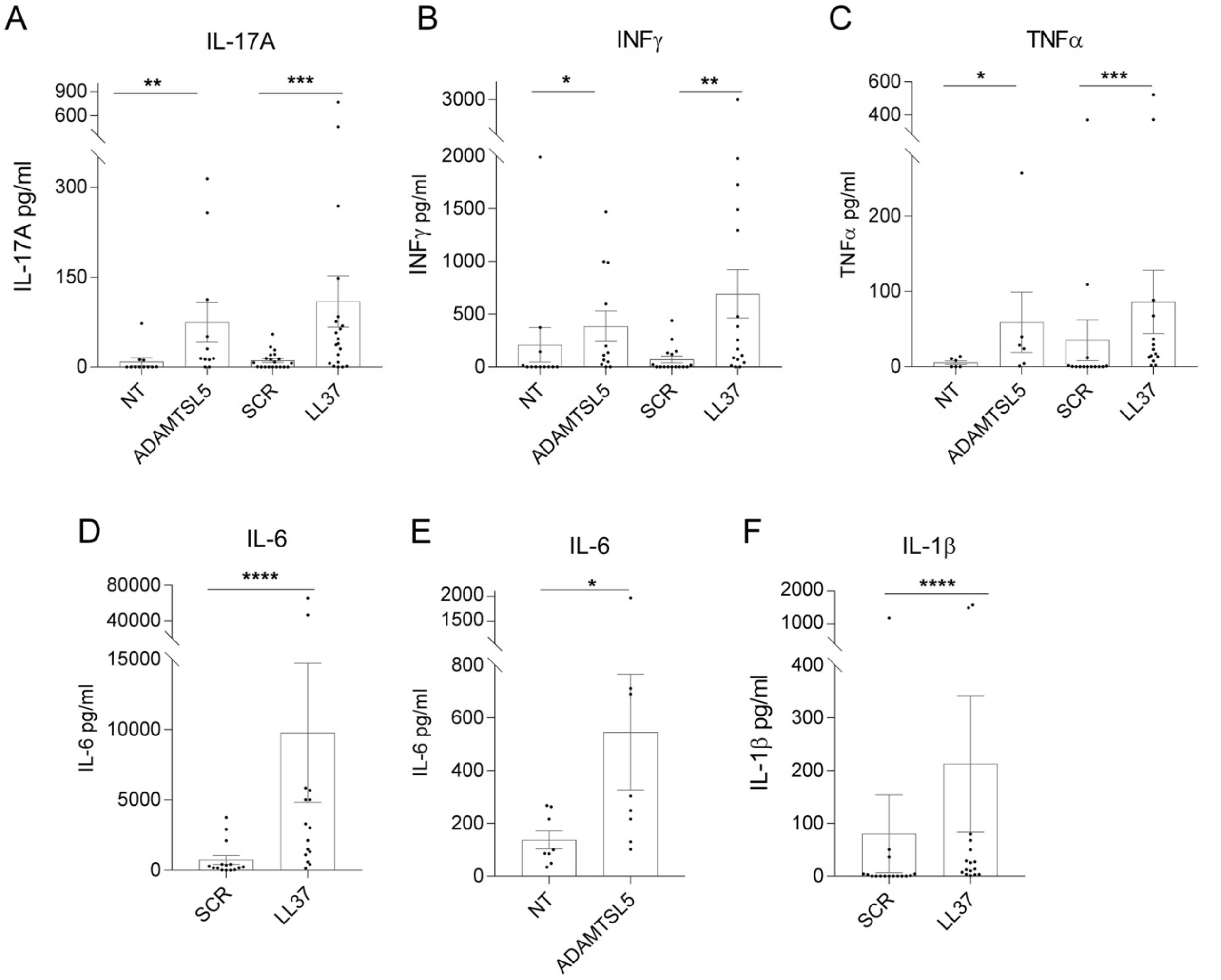

Supplementary Figure 3: Cytokine release following antigen stimulation.

Higher levels of Th17 and Th1-realted cytokines (IL-17A, IFN-γ, TNF-α) were detected in the supernatant of PBMC stimulated with the autoantigens LL37 and ADAMTSL5 compared to levels detected in the supernatant of scramble- or non-stimulated PBMCs (A, B, C). IL-6 was detected in the supernatants of PBMCs stimulated with both LL37 and ADAMTSL5 (D and E, respectively); however, IL-6 levels were markedly higher in LL37-stimulated samples. For this reason, the data are shown in two separate box plots with different y-axis scales. IL-1β was detected only in the supernatant of LL37-stimulated PBMCs (F). Statistical analysis was performed using a two-tailed Mann-Whitney test. A p-value <0.05 was considered statistically significant. * $p<0.05$; ** $p<0.01$; *** $p<0.001$; **** $p<0.0001$; ns, not significant.

Supplementary Figure 4.

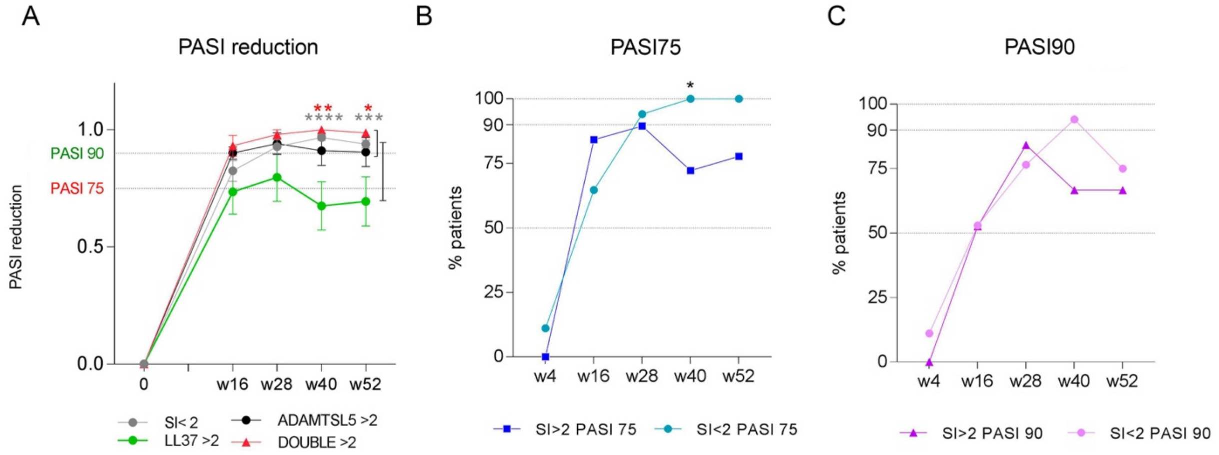

Supplementary Figure 4. PASI reduction, PASI75 and PASI90 during treatment with tildrakizumab.

A) LL37-reactive subjects demonstrated lower PASI responses at weeks 40 and 52 compared to non-reactive subjects (week 40 p<0.0001; week 52 p=0.0006, grey asterisks) and double-reactive (week 40 p=0.0049; week 52 p=0.0103, red asterisks). Non-reactive SI<2 (n=17); reactive SI>2 (n=19); LL37-reactive SI>2 (n=7); ADAMTLS5-reactive SI>2 (n=7); and double-reactive SI>2 (n=5).
B) Reactive subjects demonstrated lower PASI75 rates at week40 compared to reactive subjects.
C) No statistically significant differences were seen for PASI90, although PASI90 are numerically lower in reactive subjects compared to non-reactive subjects at week40.
Data are represented as mean and SEM, with unpaired multiple t-test with two tails. Statistical analysis for B) and C) is reporter in Suppl. Table 2. Ns, non-significant; *<0.05; **<0.01; ***<0.001.

Supplementary Table 1.

Characteristics	Psoriatic Patients n=38	Healthy donors n=33
Male (n, %)	29/38 (76.3)	17/33(51.5)
Female (n, %)	9/38 (23.7)	16/33(48.5)
Mean age (mean±sd)	48.02± 11.86	34.90± 9.75
Mean age of onset (mean±sd)	33.12± 15.02	N/A
Duration of psoriasis (mean±sd)	14.90±12.62	N/A
BMI (mean±sd)	26.47 ± 3.44	UNK
PASI at baseline (median, 25th–75th percentile).	11.5 (10-17.25)	N/A

Supplementary Table 1: Demographic and genetic characteristics of the study population.

N/A, not applicable; UNK, unknown.

Supplementary Table 2.

	Week 16		Week 28		Week 40		Week 52	
	Reactive	Non-reactive	Reactive	Non-reactive	Reactive	Non-reactive	Reactive	Non-reactive
**PASI75**	84.21 (16/19)	64.71 (11/17)	88.47 (17/19)	94.12 (16/17)	**73.68 (14/19)**	**100.00 (17/17)**	78.95 (15/19)	100.00 (17/17)
PASI90	52.63 (10/19)	52.94 (9/17)	84.21 (16/19)	76.47 (13/17)	68.42 (13/19)	94.12 (16/17)	68.42 (13/19)	76.47 (13/17)
PASI100	10.53 (2/19)	35.29 (6/17)	42.11 (8/19)	52.94 (9/17)	47.37 (9/19)	64.71 (11/17)	47.37 (9/19)	46.06 (8/17)

Supplementary Table 2: PASI75 and PASI90 responses based on reactivity status at different timepoints.

The only statistically significant difference in PASI75 or PASI90 between reactive and non-reactive subjects, was seen for PASI75 at week 40 (p=0.047). Statistically significant results are reported in bold.
